# Ophthalmia

**Published:** 1903-09-05

**Authors:** 


### Hospital Clinics and Medical Progress.

### OPHTHALMIA.

In an address delivered at the Medical Graduates' College and Polyclinic Mr. Treacher Collins [1] called attention to the fact that several different forms of inflammation of the conjunctiva are known to be connected with the growth of certain micro-organisms in the conjunctival sac. These, and some other forms in connection with which no definite micro-organisms have as yet been cultivated, are contagious, and are spread by the transmission of discharge from one person to another. The organisms, pure cultures of which have been shown to be capable of producing conjunctival inflammation, are readily killed by drying, and do not form spores. It is improbable, therefore, that dried secretion in the form of dust can prove infective, and aërial infection is therefore unlikely. Hyperæmia of the conjunctiva caused by dust or in other ways may render it more susceptible; moreover, a hyperæmic conjunctiva is more likely to be infected from the frequency with which it is rubbed by the handkerchief or fingers. It is a good working hypothesis to regard every case of ophthalmia as due to infection.

In ophthalmia neonatorum the causative agent gains access to the conjunctiva shortly after birth, when the infant first opens its eyes. At this period of life no tears are secreted and therefore an irritant introduced into the conjunctival sac is not washed away. The affection can be almost always prevented by washing the eyelids with an antiseptic lotion, removing any discharge adherent to the eyelashes, and then introducing into each eye a drop of 2 per cent. nitrate of silver solution. By the systematic adoption of this plan in lying-in establishments ophthalmia neonatorum has been practically abolished from these institutions. Kostling has collected statistics on this point; of 17,000 births before the introduction of the above treatment 9 per cent. of the babies had ophthalmia neonatorum. Of 24,000 newborn children treated as above only 0·65 per cent. developed ophthalmia. A large number of cases in which the condition occurs have been under the care of midwives, and Mr. Collins suggests that compulsory notification should be enforced of all cases occurring in the practice of others than medical men, for ophthalmia neonatorum is not only a preventable disease, but it is curable without any affection of the sight ensuing if seen and treated at a sufficiently early period. Appropriate treatment consists of the application of a 2 per cent. solution of nitrate of silver to the inner surface of the everted lids by the surgeon himself once every 24 hours, and the careful washing away of all discharge every one or two hours by the mother or nurse with a zinc chloride lotion, one grain to the ounce, or perchloride of mercury lotion, one-eighth of a grain to the ounce. In about two-thirds of the cases of ophthalmia neonatorum the gonococcus can be found in the discharge. Gonorrhœal ophthalmia in the adult is a much more severe affection than in the infant, and perforation of the cornea and destruction of the eye may ensue in spite of every effort of treatment. Prevention is therefore of the greatest importance, and any patient who is suffering from gonorrhœa should be instructed about the danger of infecting his eyes and how to avoid it. The treatment is the same as for ophthalmia neonatorum, except in the early stages, when there is considerable swelling of the lids and conjunctiva without any discharge. At this stage ice-pads should be constantly applied.

Membranous conjunctivitis may be produced by a variety of irritants, but when a case arises apart from any application to the eye, the suspicion of its being diphtheritic should at once arise. Bacterial examination alone can make the diagnosis certain. The condition is best treated with antitoxin serum. A third kind of acute inflammation of the conjunctiva is acute muco-purulent ophthalmia, with which is often associated the Koch-Weeks bacillus, and sometimes an encapsuled diplococcus which has all the appearances of the pneumococcus. The affection is highly contagious; it runs a rapid course and tends to get well of itself. The most important thing in the treatment is to keep the discharge well washed away with some weak antiseptic lotion. Care should also be taken to prevent the patient from infecting others.

One of the commonest forms of ophthalmia in this country is what Mr. Collins calls angular conjunctivitis. It is a chronic affection of the palpebral conjunctiva which, if untreated, lasts for months, the most striking feature being the marked injection of both lid margins and of the subjacent skin at the outer and inner canthus. The affection is associated with a characteristic and easily recognised bacillus, termed the diplo bacillus of Morax. The condition rapidly yields to treatment, which consists of the instillation three times a day of drops of sulphate of zinc, two grains to the ounce, and the application of boric acid ointment to the lids.

Any form of inflammation of the conjunctiva which has become chronic is liable to be accompanied by enlargement of the papillae, so that the inner surface of the lids presents a velvety appearance. One form in which the enlargement of papillae is very striking is that known as spring catarrh, which disappears during the winter and comes back again in the spring. The main symptom of which the patient complains is an intolerable itching or irritation about the eyes, and it is to the relief of this to which treatment has to be directed, as so far nothing that will cure the disease has been discovered. The condition is most commonly met with in children, and usually, after having recurred for three or four successive years, it disappears spontaneously. A lotion of carbolic acid, 1 in 40, gives most relief. In the conjunctiva there is normally some adenoid tissue, and this is liable to become increased, in which case little nodules are seen, which are most numerous in the lower *cul-de sac*. There is usually some enlargement of adenoid tissue in the naso-pharynx as well. The condition is known as follicular conjunctivitis. It is important to distinguish this condition from trachoma, as follicular conjunctivitis never leads to the formation of cicatricial tissue and other sequelæ, such as are common results of trachoma. In follicular conjunctivitis there is a simple enlargement of normal adenoid tissue; in trachoma there is extensive new formation of lymphoid tissue in situations where it is not normally present. In the latter affection the characteristic granulations are not confined to the retro-tarsal fold, but extend on to the tarsal conjunctiva. The treatment of trachoma consists of expression of the enlarged follicles with roller forceps and local applications of sulphate of copper drops. Trachoma is probably responsible for more loss of sight and more suffering than any other eye affection; its prevention is therefore an important question. There can be little doubt that the disease is contagious, and it is said to have been introduced to this country by soldiers returning from the East, where it is very prevalent, during the Napoleonic wars. In America the condition is classed among the dangerous contagious diseases, and immigrants suffering from the complaint are prohibited from entering the country.

[1] The Clinical Journal, July 29th.

